# Supplementary material for: CONFOLD2: improved contact-driven ab initio protein structure modeling
Source: BMC Bioinformatics. 2018 Jan 25;19:22. doi: 10.1186/s12859-018-2032-6 (PMC5784681; doi:10.1186/s12859-018-2032-6)
Supplement: Additional file 1 — Supplementary Tables. Docx file containing various tables with detailed results. (PDF 167 kb) [file 12859_2018_2032_MOESM1_ESM.pdf]

Supplementary Table S1. Reconstruction of the 150 proteins in the PSICOV dataset using CONFOLD2 with PSICOV and MetaPSICOV predicted contacts. Reconstruction results of the CONFOLD method are also presented for comparison. Precision of top L/5 long-range (LR) and all (long-range, medium-range, and short-range) contacts are also reported for the PSICOV and MetaPSICOV predicted contacts. CONFOLD's reconstruction accuracy is reported for 'best of 200' and 'best of 5' models for each protein.

PDB Chain	Length	CONFOLD TM-score	PSICOV Contact Precision		CONFOLD2 using PSICOV (best of 200)		CONFOLD2 using PSICOV (best of 5)		MetaPSICOV Contact Precision		CONFOLD2 using MetaPSICOV (best of 200)		CONFOLD2 using MetaPSICOV (best of 5)	
			LR - L/5	ALL - L/5	TM-score	RMSD	TM-score	RMSD	LR - L/5	ALL - L/5	TM-score	RMSD	TM-score	RMSD
1a3aA	145	0.70	86.2	89.7	0.71	4.1	0.63	4.7	96.6	100.0	0.80	3.0	0.80	3.2
1a6mA	151	0.61	56.7	56.7	0.70	4.3	0.60	5.7	86.7	80.0	0.82	2.7	0.82	2.7
1a70A	97	0.44	79.0	68.4	0.52	5.2	0.37	7.3	94.7	89.5	0.63	5.1	0.60	5.5
1aapA	56	0.49	81.8	90.9	0.50	3.7	0.39	4.4	81.8	100.0	0.57	2.7	0.50	3.5
1abaA	87	0.56	76.5	64.7	0.53	4.7	0.52	5.6	94.1	100.0	0.71	2.7	0.65	3.4
1ag6A	99	0.44	70.0	80.0	0.47	9.8	0.47	9.8	70.0	90.0	0.43	7.7	0.40	9.0
1aoeA	192	0.63	55.3	63.2	0.63	5.8	0.62	6.1	65.8	76.3	0.71	4.7	0.70	6.2
1atlA	200	0.36	50.0	55.0	0.39	9.5	0.33	13.3	65.0	70.0	0.59	8.0	0.58	8.0
1atzA	75	0.62	86.7	80.0	0.62	3.4	0.50	4.7	86.7	93.3	-	-	-	-
1avsA	81	0.68	50.0	68.8	0.63	6.4	0.48	7.2	56.3	87.5	0.59	4.3	0.52	5.9
1bdoA	80	0.52	75.0	100.0	0.56	4.2	0.53	5.7	93.8	100.0	0.57	4.2	0.52	4.3
1bebA	156	0.31	19.4	25.8	0.32	12.5	0.24	12.9	35.5	77.4	0.63	5.2	0.63	5.4
1behA	184	0.34	51.4	64.9	0.43	16.5	0.35	13.8	78.4	75.7	0.53	11.4	0.44	10.6
1bkrA	108	0.71	54.6	63.6	0.75	2.8	0.73	2.8	68.2	77.3	-	-	-	-
1brfA	53	0.37	63.6	81.8	0.38	5.7	0.37	4.8	54.6	90.9	0.46	4.2	0.41	4.8
1bsgA	266	0.74	77.4	84.9	0.78	4.3	0.77	4.4	84.9	94.3	0.69	6.0	0.68	6.1
1c44A	123	0.39	36.0	36.0	0.42	8.5	0.30	10.5	52.0	84.0	-	-	-	-
1c52A	131	0.48	53.9	42.3	0.46	11.6	0.39	13.8	61.5	61.5	-	-	-	-
1c9oA	66	0.47	69.2	92.3	0.50	3.8	0.45	4.2	84.6	92.3	0.67	2.8	0.64	2.9
1cc8A	72	0.72	100.0	85.7	0.68	3.1	0.61	5.1	92.9	85.7	0.72	2.7	0.68	2.7
1chdA	198	0.76	95.0	90.0	0.80	3.7	0.69	4.5	95.0	100.0	0.84	2.9	0.80	3.5
1cjwA	166	0.61	75.8	78.8	0.63	8.1	0.63	8.1	75.8	87.9	0.67	5.7	0.65	7.6
1ckeA	212	0.65	81.0	90.5	0.66	5.9	0.60	6.4	90.5	92.9	0.65	5.7	0.65	5.2
1ctfA	68	0.58	85.7	85.7	0.72	2.4	0.61	3.0	92.9	92.9	0.72	2.2	0.69	2.3
1cxyA	81	0.46	62.5	87.5	0.56	5.0	0.33	7.3	87.5	93.8	0.60	3.3	0.58	3.5
1cznA	169	0.60	79.4	82.4	0.56	6.1	0.55	6.3	85.3	91.2	0.67	5.4	0.62	6.0
1d0qA	102	0.62	75.0	65.0	0.73	3.7	0.63	4.2	90.0	80.0	0.75	2.9	0.72	3.5
1d1qA	159	0.75	68.8	71.9	0.76	3.5	0.69	4.1	84.4	90.6	0.80	3.2	0.79	3.6
1d4oA	177	0.41	60.0	60.0	0.40	13.4	0.32	16.1	65.7	62.9	0.55	6.5	0.55	6.5
1dbxA	152	0.58	50.0	73.3	0.64	4.6	0.46	6.4	86.7	100.0	0.80	2.9	0.72	3.9
1dixA	208	0.30	21.4	28.6	0.30	14.0	0.28	14.4	42.9	76.2	0.41	11.7	0.39	11.2
1dlwA	116	0.65	69.6	69.6	0.63	4.1	0.58	5.5	87.0	82.6	0.67	3.5	0.63	5.0
1dmgA	172	0.40	67.7	64.7	0.43	9.0	0.43	9.0	82.4	79.4	0.56	9.4	0.48	10.0
1dqgA	134	0.27	0.0	14.8	0.27	11.0	0.24	11.8	11.1	88.9	0.26	16.4	0.25	13.8
1dsxA	87	0.37	52.9	41.2	0.49	5.2	0.46	5.5	64.7	82.4	0.67	3.3	0.60	3.9
1eazA	103	0.71	76.2	95.2	0.74	2.7	0.70	3.3	71.4	100.0	0.74	3.3	0.63	4.1
1ej0A	180	0.59	55.6	66.7	0.60	6.0	0.60	6.0	91.7	94.4	0.78	3.6	0.78	3.4
1ej8A	140	0.29	35.7	53.6	0.30	17.1	0.24	23.7	57.1	82.1	0.40	9.8	0.37	9.0
1ek0A	168	0.74	67.7	67.7	0.77	3.1	0.75	3.6	91.2	91.2	0.79	3.1	0.75	3.8
1f6bA	176	0.55	71.4	65.7	0.56	8.7	0.51	9.3	88.6	91.4	0.62	7.9	0.60	9.1
1fcyA	236	0.33	27.7	34.0	0.41	29.5	0.37	19.8	40.4	68.1	0.45	22.4	0.42	14.6
1fk5A	93	0.39	26.3	26.3	0.42	9.6	0.39	9.9	63.2	47.4	0.60	6.6	0.58	5.2
1fl0A	164	0.42	51.5	75.8	0.37	18.1	0.36	11.3	72.7	81.8	0.47	9.3	0.41	10.1
1fnaA	91	0.60	83.3	77.8	0.58	4.6	0.51	9.2	88.9	88.9	0.68	3.7	0.66	3.7
1fqtA	109	0.68	81.8	81.8	0.68	6.1	0.59	5.9	90.9	100.0	0.73	3.3	0.73	3.5
1fvgA	192	0.63	84.2	86.8	0.66	5.4	0.65	6.4	86.8	86.8	0.69	5.1	0.68	5.6
1fvkA	188	0.42	73.7	73.7	0.52	11.5	0.52	11.5	73.7	79.0	0.56	11.8	0.56	11.8
1fx2A	112	0.43	77.3	81.8	0.50	11.7	0.47	14.3	81.8	86.4	0.56	10.8	0.47	10.6
1g2rA	94	0.58	47.4	79.0	0.49	7.1	0.45	6.7	73.7	89.5	0.73	4.1	0.70	3.4
1g9oA	91	0.57	94.4	94.4	0.55	6.4	0.52	7.0	88.9	94.4	0.58	5.5	0.52	5.5
1gbsA	185	0.48	73.0	86.5	0.48	18.2	0.48	20.8	86.5	89.2	0.57	17.9	0.55	19.7
1gmiA	135	0.51	81.5	96.3	0.48	13.8	0.42	9.6	92.6	92.6	0.64	6.8	0.62	6.7
1gmxA	107	0.66	85.7	81.0	0.71	4.9	0.65	4.2	85.7	90.5	0.78	2.7	0.74	3.5
1guuA	50	0.68	60.0	70.0	0.62	3.2	0.55	5.6	70.0	90.0	0.68	3.8	0.64	2.9

PDB Chain	Length	CONFOLD TM-score	PSICOV Contact Precision		CONFOLD2 using PSICOV (best of 200)		CONFOLD2 using PSICOV (best of 5)		MetaPSICOV Contact Precision		CONFOLD2 using MetaPSICOV (best of 200)		CONFOLD2 using MetaPSICOV (best of 5)	
			LR - L/5	ALL - L/5	TM-score	RMSD	TM-score	RMSD	LR - L/5	ALL - L/5	TM-score	RMSD	TM-score	RMSD
1gz2A	138	0.64	60.7	67.9	0.67	4.0	0.59	5.8	82.1	89.3	0.71	4.8	0.68	4.3
1gzcA	239	0.33	50.0	60.4	0.33	13.0	0.28	14.5	62.5	83.3	0.50	10.9	0.50	10.9
1h0pA	182	0.69	69.4	86.1	0.71	5.9	0.66	10.8	75.0	94.4	0.73	6.8	0.73	9.5
1h2eA	207	0.82	85.4	87.8	0.83	3.8	0.82	3.7	100.0	100.0	0.84	4.3	0.82	4.2
1h4xA	110	0.65	54.6	59.1	0.61	4.5	0.59	4.1	90.9	90.9	0.72	3.0	0.66	3.2
1h98A	77	0.40	33.3	66.7	0.42	8.4	0.39	8.1	40.0	80.0	0.44	10.3	0.39	10.7
1hdoA	205	0.75	85.4	92.7	0.77	5.1	0.74	6.4	92.7	95.1	0.78	4.9	0.76	4.7
1hfcA	157	0.46	54.8	64.5	0.53	8.1	0.53	8.1	67.7	61.3	0.59	5.2	0.55	5.9
1hh8A	192	0.40	52.6	81.6	0.43	41.5	0.38	21.6	65.8	100.0	0.51	10.6	0.42	22.2
1htwA	158	0.66	81.3	84.4	0.68	4.8	0.68	4.8	87.5	96.9	0.75	3.2	0.70	3.9
1hxnA	210	0.26	11.9	52.4	0.27	15.2	0.22	16.5	16.7	88.1	0.41	10.9	0.34	16.6
1i1jA	106	0.26	33.3	66.7	0.34	16.2	0.21	13.4	38.1	90.5	0.41	11.4	0.35	12.1
1i1nA	224	0.69	71.1	80.0	0.71	5.7	0.65	6.5	77.8	95.6	0.69	5.7	0.66	5.5
1i4jA	110	0.53	77.3	81.8	0.58	9.0	0.57	9.6	100.0	100.0	0.63	6.3	0.62	7.1
1i58A	189	0.61	57.9	73.7	0.62	6.8	0.58	6.6	71.1	92.1	0.73	4.3	0.69	5.0
1i5gA	144	0.50	72.4	58.6	0.53	8.8	0.48	9.1	93.1	82.8	0.61	6.2	0.53	7.7
1i71A	83	0.36	58.8	76.5	0.37	8.9	0.31	8.6	76.5	88.2	0.45	5.2	0.40	5.8
1ihzA	136	0.66	70.4	77.8	0.68	4.4	0.68	4.1	77.8	100.0	0.70	3.9	0.67	4.3
1iibA	103	0.63	71.4	85.7	0.68	3.1	0.68	3.1	95.2	95.2	0.84	1.8	0.81	2.0
1im5A	179	0.59	80.6	80.6	0.61	6.9	0.58	6.7	100.0	100.0	0.72	5.5	0.72	5.4
1iwdA	215	0.72	83.7	83.7	0.72	4.7	0.68	5.8	83.7	86.1	0.72	5.4	0.67	5.8
1j3aA	129	0.55	80.8	80.8	0.55	9.5	0.44	16.0	88.5	80.8	0.63	7.1	0.58	7.8
1jbeA	126	0.83	80.0	84.0	0.83	2.5	0.81	2.8	96.0	96.0	0.83	2.4	0.80	2.6
1jbkA	189	0.32	34.2	31.6	0.31	13.3	0.31	13.0	73.7	68.4	0.69	4.6	0.64	5.4
1jfuA	176	0.62	82.9	77.1	0.60	10.2	0.58	11.5	91.4	97.1	0.62	13.0	0.62	11.5
1jfxA	217	0.66	62.8	74.4	0.68	5.5	0.56	7.1	86.1	88.4	0.72	4.5	0.69	5.1
1jkxA	209	0.68	66.7	71.4	0.69	8.0	0.65	8.0	90.5	97.6	0.77	6.8	0.75	4.3
1jl1A	152	0.69	76.7	80.0	0.70	8.3	0.68	6.4	90.0	96.7	0.73	5.5	0.67	9.2
1jo0A	97	0.44	47.4	52.6	0.51	10.9	0.38	12.4	63.2	89.5	0.65	3.6	0.61	6.4
1jo8A	58	0.55	83.3	100.0	0.60	3.0	0.59	3.6	83.3	100.0	0.66	2.7	0.63	2.8
1josA	100	0.62	80.0	85.0	0.62	3.8	0.56	4.8	85.0	85.0	0.72	3.7	0.68	3.2
1jvwA	160	0.57	78.1	93.8	0.58	10.7	0.54	11.8	93.8	96.9	0.66	12.2	0.66	12.2
1jwqA	179	0.79	91.7	88.9	0.83	2.6	0.81	2.7	97.2	97.2	0.86	2.3	0.85	2.4
1jyhA	155	0.36	51.6	71.0	0.36	9.9	0.34	9.7	80.7	90.3	0.55	5.4	0.51	7.0
1k6kA	142	0.66	75.0	82.1	0.75	3.0	0.70	4.3	85.7	92.9	0.80	3.0	0.73	3.5
1k7cA	233	0.52	76.6	80.9	0.55	10.8	0.51	12.1	93.6	95.7	0.68	8.4	0.68	9.6
1k7jA	206	0.63	68.3	73.2	0.59	8.8	0.55	10.4	85.4	95.1	0.74	4.0	0.74	4.0
1kidA	193	0.41	48.7	71.8	0.44	8.0	0.39	9.5	82.1	92.3	0.62	6.4	0.54	6.4
1kq6A	140	0.39	35.7	57.1	0.49	17.0	0.48	23.5	50.0	75.0	0.53	14.2	0.46	15.8
1kqrA	160	0.23	15.6	28.1	0.22	14.3	0.21	14.3	18.8	68.8	0.31	14.4	0.29	14.2
1ktgA	137	0.59	59.3	66.7	0.57	8.7	0.57	6.8	74.1	81.5	0.70	4.0	0.67	4.1
1ku3A	61	0.66	58.3	83.3	0.76	4.9	0.71	2.8	66.7	83.3	0.77	2.0	0.68	3.7
1kw4A	70	0.52	64.3	85.7	0.51	4.2	0.51	4.2	64.3	71.4	0.73	3.3	0.65	2.5
1lm4A	189	0.64	71.1	76.3	0.63	9.4	0.59	9.6	65.8	86.8	0.66	9.7	0.63	9.6
1lo7A	140	0.57	85.7	82.1	0.60	8.9	0.54	9.7	96.4	100.0	0.72	5.3	0.72	6.8
1lpyA	162	0.57	56.3	71.9	0.55	6.7	0.50	6.9	59.4	71.9	0.52	6.5	0.52	7.6
1m4jA	133	0.33	33.3	37.0	0.37	8.2	0.29	9.7	74.1	77.8	0.60	5.9	0.60	4.6
1m8aA	61	0.50	33.3	66.7	0.53	4.3	0.53	4.3	50.0	83.3	0.69	2.3	0.62	2.9
1mk0A	97	0.39	57.9	73.7	0.49	7.3	0.43	6.2	79.0	84.2	0.49	7.8	0.32	9.9
1mugA	165	0.38	45.5	51.5	0.38	9.4	0.35	10.2	93.9	90.9	0.69	5.9	0.66	6.3
1nb9A	147	0.62	69.0	79.3	0.65	10.2	0.58	9.2	86.2	82.8	0.67	8.0	0.65	8.9
1ne2A	176	0.46	54.3	82.9	0.43	12.9	0.42	12.9	77.1	91.4	0.55	11.0	0.55	7.8
1npsA	88	0.51	66.7	66.7	0.44	7.0	0.42	6.1	77.8	100.0	0.51	5.1	0.46	5.8
1nrvA	100	0.62	45.0	75.0	0.57	7.8	0.53	7.5	65.0	100.0	0.64	6.3	0.57	6.3
1ny1A	235	0.53	80.9	78.7	0.52	18.2	0.51	21.1	91.5	87.2	0.61	14.4	0.56	14.4
1o1zA	226	0.64	75.6	75.6	0.68	6.9	0.64	7.6	84.4	93.3	0.79	3.3	0.77	3.5
1p90A	123	0.49	56.0	60.0	0.57	4.7	0.55	6.7	68.0	84.0	0.61	6.8	0.61	6.8
1pchA	88	0.69	100.0	94.4	0.75	2.7	0.75	2.7	83.3	77.8	0.79	2.9	0.77	3.1
1pkoA	124	0.55	80.0	76.0	0.53	8.0	0.51	9.2	96.0	92.0	0.62	8.5	0.61	8.4
1qf9A	194	0.69	66.7	66.7	0.66	4.8	0.61	7.2	82.1	87.2	0.75	4.0	0.69	4.5

PDB Chain	Length	CONFOLD TM-score	PSICOV Contact Precision		CONFOLD2 using PSICOV (best of 200)		CONFOLD2 using PSICOV (best of 5)		MetaPSICOV Contact Precision		CONFOLD2 using MetaPSICOV (best of 200)		CONFOLD2 using MetaPSICOV (best of 5)	
			LR - L/5	ALL - L/5	TM-score	RMSD	TM-score	RMSD	LR - L/5	ALL - L/5	TM-score	RMSD	TM-score	RMSD
1qjpA	137	0.56	59.3	88.9	0.58	6.8	0.46	8.8	70.4	96.3	0.60	5.4	0.58	6.2
1ql0A	241	0.39	68.8	72.9	0.36	14.8	0.34	15.2	72.9	85.4	0.54	11.1	0.52	12.4
1r26A	113	0.74	82.6	69.6	0.73	4.9	0.73	6.1	100.0	95.7	0.82	3.6	0.76	4.6
1roaA	111	0.41	36.4	63.6	0.41	12.0	0.40	9.6	59.1	77.3	0.47	8.0	0.44	7.1
1rw1A	114	0.69	56.5	56.5	0.67	3.5	0.59	4.1	95.7	91.3	0.80	2.2	0.76	2.8
1rw7A	235	0.60	83.0	74.5	0.62	9.0	0.60	9.4	93.6	97.9	0.72	5.4	0.67	5.9
1rybA	186	0.72	75.7	86.5	0.71	4.3	0.68	4.7	94.6	97.3	0.80	3.4	0.79	3.5
1smxA	87	0.28	47.1	70.6	0.35	11.0	0.28	13.0	58.8	82.4	0.48	7.4	0.47	6.8
1svyA	101	0.41	35.0	45.0	0.47	8.3	0.43	9.3	75.0	95.0	0.64	7.4	0.57	7.6
1t8kA	77	0.65	86.7	80.0	0.66	3.4	0.62	3.8	86.7	93.3	0.79	2.3	0.69	2.7
1tifA	76	0.52	33.3	93.3	0.54	3.8	0.49	6.1	53.3	93.3	0.63	3.9	0.57	3.6
1tqgA	105	0.77	57.1	71.4	0.85	1.9	0.77	2.4	76.2	95.2	0.87	1.9	0.86	2.3
1tqhA	242	0.67	75.0	81.3	0.72	7.3	0.71	9.0	97.9	100.0	0.74	8.0	0.73	10.4
1tzvA	141	0.74	57.1	67.9	0.76	3.5	0.71	4.1	82.1	96.4	0.83	2.8	0.80	2.7
1vfyA	67	0.37	46.2	69.2	0.39	6.9	0.33	7.2	46.2	76.9	0.38	6.8	0.34	6.3
1vhuA	192	0.67	71.1	76.3	0.67	8.2	0.67	8.2	84.2	86.8	0.78	3.3	0.77	3.9
1vjkA	87	0.61	70.6	88.2	0.61	4.1	0.51	5.3	94.1	94.1	0.71	5.0	0.69	4.2
1vmbA	107	0.55	81.0	81.0	0.55	6.0	0.42	6.4	81.0	85.7	0.70	4.1	0.66	5.7
1vp6A	133	0.72	100.0	96.3	0.73	4.4	0.68	4.9	96.3	100.0	0.73	3.2	0.71	3.6
1w0hA	200	0.70	77.5	82.5	0.72	7.8	0.68	7.4	87.5	95.0	0.76	6.3	0.73	8.2
1whiA	122	0.47	66.7	91.7	0.44	7.2	0.38	8.1	79.2	91.7	0.44	7.5	0.41	8.4
1wjxA	112	0.60	77.3	81.8	0.59	4.3	0.58	4.5	72.7	95.5	0.71	3.4	0.64	4.0
1wkcA	168	0.68	58.8	64.7	0.76	3.5	0.67	4.1	76.5	91.2	0.81	3.1	0.78	3.4
1xdzA	238	0.54	75.0	81.3	0.58	7.3	0.56	7.6	91.7	97.9	0.70	5.0	0.59	9.8
1xffA	238	0.69	75.0	89.6	0.69	5.5	0.67	5.5	95.8	97.9	0.78	4.4	0.77	4.0
1xkrA	205	0.41	26.8	31.7	0.35	10.1	0.33	12.6	26.8	63.4	0.44	14.4	0.38	13.1
2arcA	161	0.29	46.9	34.4	0.29	16.3	0.24	21.0	62.5	75.0	0.40	11.6	0.35	11.7
2cuaA	122	0.44	79.2	95.8	0.48	13.8	0.46	15.3	95.8	95.8	0.60	9.7	0.58	8.4
2hs1A	99	0.38	35.0	60.0	0.38	8.0	0.34	9.2	20.0	70.0	0.32	10.2	0.27	11.4
2mhrA	118	0.66	54.2	70.8	0.66	5.6	0.64	6.6	66.7	79.2	0.76	5.0	0.76	4.8
2phyA	125	0.39	20.0	76.0	0.39	15.5	0.39	15.5	28.0	92.0	0.46	8.1	0.46	8.1
2tpsA	226	0.76	71.1	66.7	0.73	6.8	0.61	13.3	84.4	93.3	0.82	5.4	0.80	6.4
2vxnA	249	0.75	68.0	78.0	0.76	4.6	0.66	5.9	94.0	94.0	0.86	3.3	0.82	3.7
3borA	194	0.76	87.2	84.6	0.76	4.0	0.72	4.1	94.9	89.7	0.82	3.3	0.77	3.5
3dqgA	148	0.46	73.3	86.7	0.55	10.2	0.52	10.5	83.3	96.7	0.60	9.8	0.60	9.8
5ptpA	222	0.82	88.6	95.5	0.81	3.0	0.81	3.3	95.5	95.5	0.85	2.7	0.82	2.9
**Avg**	**146**	**0.55**	**64.0**	**72.7**	**0.57**	**7.9**	**0.52**	**8.4**	**77.2**	**88.4**	**0.65**	**6.1**	**0.62**	**6.5**

Supplementary Table S2. Reconstruction of the CASP11 regular target proteins using CONFOLD2 with CONSIP2 predicted contacts. Precision of top L/5 long-range (LR) and all (long-range, medium-range, and short-range) contacts are also reported. CONFOLD's reconstruction accuracy is reported for 'best of 200' and 'best of 5' models for each protein.

Domain	Target	L_{target}	Contact Precision Top L/5		Best of 200 (Models)			Best of 5 (Models)		
			SR + MR + LR	Long-range	TM-score	RMSD	GDT-TS	TM-score	RMSD	GDT-TS
T0759-D1	T0759	109	42.86	14.29	0.51	3.66	75.00	0.35	4.18	58.82
T0759-D2	T0759	109	83.33	8.33	0.36	8.81	44.35	0.31	12.35	35.08
T0760-D1	T0760	242	85.00	60.00	0.33	13.35	19.53	0.31	14.68	18.41
T0761-D1	T0761	285	66.67	5.56	0.29	12.96	28.69	0.23	11.68	23.58
T0761-D2	T0761	285	78.26	8.70	0.30	15.29	28.54	0.23	20.61	21.02
T0762-D1	T0762	280	88.24	90.20	0.55	8.85	35.99	0.49	12.60	30.64
T0763-D1	T0763	163	80.77	46.15	0.27	16.90	21.73	0.24	17.61	20.58
T0764-D1	T0764	341	95.31	87.50	0.61	16.87	40.81	0.58	13.10	39.72
T0765-D1	T0765	128	80.00	80.00	0.70	2.74	70.72	0.52	4.34	57.57
T0766-D1	T0766	130	90.91	22.73	0.44	6.24	43.06	0.28	11.78	29.17
T0767-D1	T0767	318	86.67	33.33	0.45	7.78	50.66	0.38	10.44	44.41
T0767-D2	T0767	318	25.00	58.33	0.32	18.32	22.36	0.32	17.72	23.33
T0768-D1	T0768	170	89.66	48.28	0.52	10.55	41.96	0.51	9.69	41.61
T0769-D1	T0769	112	84.21	21.05	0.51	4.59	51.80	0.44	6.23	45.10
T0770-D1	T0770	488	78.02	76.92	0.58	12.53	30.98	0.52	13.59	28.89

Domain	Target	L_{target}	Contact Precision Top L/5		Best of 200 (Models)			Best of 5 (Models)		
			SR + MR + LR	Long-range	TM-score	RMSD	GDT-TS	TM-score	RMSD	GDT-TS
T0771-D1	T0771	204	53.33	10.00	0.26	15.73	18.87	0.24	17.74	19.21
T0772-D1	T0772	265	83.72	83.72	0.53	8.49	35.28	0.52	8.90	33.29
T0773-D1	T0773	77	92.31	23.08	0.53	4.53	58.96	0.48	5.04	54.85
T0774-D1	T0774	379	70.15	62.69	0.29	19.80	13.21	0.29	19.80	13.21
T0776-D1	T0776	256	93.18	93.18	0.71	7.08	56.16	0.71	7.08	56.16
T0777-D1	T0777	366	52.17	23.19	0.39	15.16	19.71	0.39	15.16	19.71
T0780-D1	T0780	259	94.74	84.21	0.34	8.30	34.47	0.27	11.53	27.11
T0780-D2	T0780	259	94.74	73.68	0.48	4.82	46.61	0.41	7.12	39.06
T0781-D1	T0781	420	55.00	5.00	0.24	19.10	17.00	0.23	21.73	15.87
T0781-D2	T0781	420	77.14	28.57	0.28	14.72	19.86	0.27	16.75	22.57
T0782-D1	T0782	135	81.82	31.82	0.47	7.51	43.18	0.43	10.89	38.86
T0783-D1	T0783	411	87.76	89.80	0.61	9.11	41.46	0.60	9.04	39.51
T0783-D2	T0783	411	32.26	51.61	0.28	18.05	21.63	0.23	42.83	20.83
T0784-D1	T0784	155	52.00	8.00	0.30	12.90	23.00	0.28	17.02	22.80
T0785-D1	T0785	115	50.00	18.18	0.30	12.37	25.22	0.27	13.49	23.44
T0786-D1	T0786	264	74.42	62.79	0.64	5.43	44.35	0.62	5.96	42.86
T0789-D1	T0789	295	75.86	51.72	0.37	11.38	29.72	0.31	15.06	26.92
T0789-D2	T0789	295	88.00	28.00	0.47	9.91	39.68	0.34	11.44	29.56
T0790-D1	T0790	293	81.48	44.44	0.58	6.47	48.52	0.33	12.62	28.33
T0790-D2	T0790	293	84.62	26.92	0.43	7.41	38.65	0.24	17.15	20.96
T0791-D1	T0791	300	83.33	53.33	0.46	9.98	38.76	0.30	15.18	24.16
T0791-D2	T0791	300	78.57	42.86	0.39	9.63	31.16	0.30	12.08	25.18
T0792-D1	T0792	80	87.50	18.75	0.42	8.31	43.91	0.38	8.86	41.35
T0794-D1	T0794	470	94.83	93.10	0.63	8.68	40.54	0.62	6.94	37.93
T0794-D2	T0794	470	47.06	26.47	0.32	13.01	20.64	0.24	19.87	15.55
T0796-D1	T0796	309	45.76	30.51	0.18	40.68	8.70	0.16	29.84	8.95
T0800-D1	T0800	247	57.14	66.67	0.38	9.87	22.29	0.34	11.81	18.87
T0801-D1	T0801	376	98.61	95.83	0.74	6.24	46.46	0.70	7.11	41.74
T0803-D1	T0803	139	59.26	18.52	0.35	14.04	32.09	0.23	14.26	19.96
T0805-D1	T0805	214	82.05	87.18	0.66	12.28	51.52	0.65	12.51	50.89
T0806-D1	T0806	258	80.39	84.31	0.75	4.04	50.29	0.72	4.34	49.61
T0807-D1	T0807	284	94.74	94.74	0.83	5.94	61.13	0.80	6.89	58.04
T0808-D1	T0808	418	50.00	19.23	0.35	11.81	26.15	0.29	12.66	22.14
T0808-D2	T0808	418	77.78	35.19	0.33	18.08	16.82	0.28	18.83	14.59
T0810-D1	T0810	383	0.00	17.39	0.20	26.27	19.25	0.16	30.53	17.48
T0810-D2	T0810	383	84.44	84.44	0.60	11.01	42.22	0.60	11.01	42.22
T0811-D1	T0811	255	94.00	90.00	0.87	2.96	68.03	0.85	3.50	64.74
T0812-D1	T0812	204	58.33	63.89	0.39	15.64	26.92	0.28	13.45	17.58
T0813-D1	T0813	307	91.67	88.33	0.54	12.51	34.35	0.54	15.26	36.42
T0814-D1	T0814	424	74.07	37.04	0.28	13.68	21.17	0.22	14.17	16.42
T0814-D2	T0814	424	95.65	82.61	0.37	9.86	34.05	0.30	11.68	24.78
T0814-D3	T0814	424	65.52	62.07	0.32	13.82	22.57	0.27	14.16	19.44
T0815-D1	T0815	106	100.00	85.71	0.77	2.53	71.46	0.76	2.84	68.63
T0816-D1	T0816	68	28.57	7.14	0.45	4.79	51.47	0.38	8.89	47.43
T0817-D1	T0817	525	96.23	88.68	0.68	5.70	44.72	0.66	5.73	42.83
T0817-D2	T0817	525	92.86	85.71	0.71	4.56	53.33	0.71	5.25	53.81
T0818-D1	T0818	166	74.07	59.26	0.32	13.26	26.87	0.28	15.16	24.07
T0819-D1	T0819	373	98.63	95.89	0.76	6.54	48.91	0.72	6.24	43.66
T0820-D1	T0820	140	16.67	5.56	0.34	12.30	35.56	0.29	13.49	31.11
T0820-D2	T0820	140	71.43	-	0.34	4.60	56.25	0.26	6.28	48.61
T0821-D1	T0821	275	98.04	92.16	0.67	6.91	51.76	0.54	9.81	36.96
T0822-D1	T0822	121	56.52	52.17	0.35	10.74	29.17	0.35	10.74	29.17
T0823-D1	T0823	296	84.48	89.66	0.78	4.22	54.25	0.75	4.72	50.52
T0824-D1	T0824	110	63.64	45.45	0.48	6.84	43.98	0.47	5.99	42.36
T0827-D1	T0827	407	69.23	58.97	0.43	14.12	31.74	0.31	14.28	20.21
T0827-D2	T0827	407	50.00	10.00	0.34	15.31	26.50	0.27	15.71	22.33
T0829-D1	T0829	70	84.62	84.62	0.72	2.48	74.63	0.72	2.48	74.63
T0830-D1	T0830	575	81.93	74.70	0.45	25.43	22.90	0.44	19.23	21.88
T0830-D2	T0830	575	45.45	31.82	0.32	10.39	27.70	0.23	12.11	20.95
T0831-D1	T0831	419	45.16	48.39	0.48	15.24	42.42	0.40	19.55	32.26
T0831-D2	T0831	419	28.21	7.69	0.30	47.95	25.25	0.24	27.90	20.30

Domain	Target	L_{target}	Contact Precision Top L/5		Best of 200 (Models)			Best of 5 (Models)		
			SR + MR + LR	Long-range	TM-score	RMSD	GDT-TS	TM-score	RMSD	GDT-TS
T0832-D1	T0832	257	42.86	2.38	0.29	15.86	18.90	0.25	20.26	16.75
T0833-D1	T0833	136	95.45	18.18	0.50	7.72	45.83	0.50	7.53	43.29
T0834-D1	T0834	219	90.00	5.00	0.29	19.75	29.04	0.23	19.69	24.24
T0834-D2	T0834	219	47.06	17.65	0.42	9.06	42.15	0.29	9.55	32.27
T0835-D1	T0835	424	80.25	51.85	0.48	12.41	25.25	0.44	13.76	22.15
T0836-D1	T0836	204	46.34	43.90	0.53	7.06	36.03	0.51	8.46	36.76
T0837-D1	T0837	128	33.33	29.17	0.40	8.23	35.33	0.34	8.84	32.44
T0838-D1	T0838	154	40.00	12.00	0.35	10.60	29.17	0.30	12.78	25.99
T0840-D1	T0840	669	82.69	40.38	0.29	20.47	8.72	0.26	22.55	7.71
T0840-D2	T0840	669	5.56	38.89	0.25	14.79	23.10	0.20	14.11	19.02
T0841-D1	T0841	253	97.83	89.13	0.73	8.96	53.90	0.70	10.45	49.89
T0843-D1	T0843	369	85.14	87.84	0.70	9.73	44.99	0.69	10.27	43.02
T0845-D1	T0845	448	63.16	26.32	0.29	15.00	29.38	0.29	15.00	29.38
T0845-D2	T0845	448	84.85	37.88	0.36	13.91	14.06	0.29	17.43	12.39
T0847-D1	T0847	176	91.18	88.24	0.73	5.55	61.83	0.73	6.03	61.98
T0848-D1	T0848	354	60.71	46.43	0.26	16.25	18.66	0.22	17.97	15.94
T0848-D2	T0848	354	70.27	2.70	0.29	31.87	22.54	0.23	23.08	18.31
T0849-D1	T0849	240	74.47	65.96	0.59	9.36	40.15	0.50	12.85	34.53
T0851-D1	T0851	456	91.21	87.91	0.49	13.87	25.83	0.40	19.13	22.30
T0852-D1	T0852	414	87.23	89.36	0.63	5.90	41.67	0.58	7.99	37.61
T0852-D2	T0852	414	72.00	40.00	0.44	9.69	36.71	0.28	17.32	25.79
T0853-D1	T0853	152	80.00	33.33	0.33	8.55	37.50	0.23	14.22	29.28
T0853-D2	T0853	152	50.00	28.57	0.36	9.04	38.89	0.32	9.32	35.76
T0854-D1	T0854	212	100.00	100.00	0.79	2.80	69.51	0.76	2.82	65.72
T0854-D2	T0854	212	57.14	35.71	0.56	3.53	62.14	0.47	4.63	53.21
T0855-D1	T0855	119	73.91	17.39	0.40	12.13	35.43	0.32	11.61	30.22
T0856-D1	T0856	159	75.00	78.12	0.49	10.00	37.74	0.46	10.86	33.81
T0857-D1	T0857	105	57.89	47.37	0.43	9.27	40.63	0.33	8.03	32.55
T0858-D1	T0858	494	93.33	78.89	0.61	10.16	31.94	0.61	9.83	31.44
**Average**			**71.86**	**50.18**	**0.46**	**11.44**	**37.11**	**0.41**	**12.71**	**32.73**

Supplementary Table S3. Reconstruction of the CASP12 regular target proteins using CONFOLD2 with RaptorX predicted contacts. Precision of top L/5 long-range (LR) and all (long-range, medium-range, and short-range) contacts are also reported. CONFOLD's reconstruction accuracy is reported for 'best of 200' and 'best of 5' models for each protein.

Domain	Target	L_{target}	Contact Precision Top L/5		Best of 200 (Models)			Best of 5 (Models)		
			SR + MR + LR	Long-range	TM-score	RMSD	GDTTS	TM-score	RMSD	GDTTS
T0859-D1	T0859	133	0.00	0.00	0.27	13.81	24.78	0.22	15.72	21.90
T0860-D1	T0860	137	92.59	33.33	0.28	13.47	22.61	0.25	14.78	18.01
T0861-D1	T0861	323	91.94	95.16	0.82	3.83	61.06	0.79	4.47	56.01
T0862-D1	T0862	239	57.89	42.11	0.53	9.29	54.30	0.47	12.34	46.24
T0863-D1	T0863	670	30.77	28.21	0.29	18.56	20.85	0.22	18.55	14.77
T0863-D2	T0863	670	25.35	23.94	0.25	32.47	14.61	0.20	33.35	10.88
T0864-D1	T0864	246	100.00	79.59	0.35	15.51	20.63	0.30	15.90	16.57
T0865-D1	T0865	75	8.33	-	0.49	16.86	58.47	0.46	16.84	52.02
T0866-D1	T0866	183	95.24	95.24	0.63	5.02	58.89	0.60	5.00	57.93
T0867-D1	T0867	104	19.05	14.29	0.29	13.57	26.68	0.28	13.34	25.00
T0868-D1	T0868	161	43.48	56.52	0.34	9.56	28.66	0.30	12.20	26.51
T0869-D1	T0869	120	61.90	57.14	0.54	7.89	49.52	0.49	8.11	44.71
T0870-D1	T0870	138	24.00	12.00	0.40	9.64	35.37	0.27	12.00	24.80
T0871-D1	T0871	375	82.81	89.06	0.52	16.63	29.47	0.41	17.37	22.02
T0872-D1	T0872	91	88.89	77.78	0.50	6.66	49.15	0.37	8.22	39.77
T0873-D1	T0873	501	82.61	90.22	0.49	16.00	26.35	0.46	15.97	19.97
T0877-D1	T0877	142	57.14	60.71	0.32	9.72	25.70	0.27	12.12	20.25
T0878-D1	T0878	358	59.42	24.64	0.38	15.44	17.30	0.33	17.26	15.48
T0879-D1	T0879	223	86.36	93.18	0.70	11.42	53.52	0.66	10.72	46.70
T0880-D1	T0880	193	9.68	-	0.24	9.12	37.50	0.24	9.12	37.50
T0880-D2	T0880	193	71.43	19.35	0.26	15.73	17.99	0.23	16.58	15.61
T0881-D1	T0881	202	82.50	10.00	0.27	15.97	15.10	0.24	17.65	13.86
T0882-D1	T0882	89	87.50	6.25	0.40	8.64	39.24	0.34	9.97	35.44

Domain	Target	L_{target}	Contact Precision Top L/5		Best of 200 (Models)			Best of 5 (Models)		
			SR + MR + LR	Long-range	TM-score	RMSD	GDTTS	TM-score	RMSD	GDTTS
T0884-D1	T0884	75	78.57	14.29	0.31	15.57	34.51	0.24	12.89	27.11
T0885-D1	T0885	116	78.26	78.26	0.70	6.08	63.82	0.68	5.82	62.06
T0886-D1	T0886	346	78.57	100.00	0.50	4.46	57.25	0.47	4.70	50.72
T0886-D2	T0886	346	92.00	92.00	0.51	6.04	42.32	0.38	8.10	30.71
T0888-D1	T0888	121	25.00	25.00	0.22	16.21	16.74	0.20	16.11	15.70
T0889-D1	T0889	242	93.75	97.92	0.82	3.62	63.81	0.80	3.95	61.51
T0890-D1	T0890	191	81.25	68.75	0.56	6.69	57.93	0.49	7.94	50.61
T0890-D2	T0890	191	52.38	19.05	0.41	6.95	37.74	0.35	7.34	35.38
T0891-D1	T0891	130	86.36	95.45	0.68	3.98	61.61	0.65	4.08	59.82
T0892-D1	T0892	193	35.71	50.00	0.65	2.88	70.29	0.56	3.34	62.32
T0892-D2	T0892	193	63.64	72.73	0.44	10.57	39.77	0.40	10.12	35.68
T0893-D1	T0893	242	40.00	20.00	0.59	4.52	62.67	0.54	4.44	60.62
T0893-D2	T0893	242	88.24	97.06	0.79	5.38	69.08	0.77	5.89	66.42
T0894-D1	T0894	324	33.33	5.56	0.27	12.14	27.25	0.21	12.57	22.47
T0894-D2	T0894	324	100.00	0.00	0.41	8.99	47.69	0.41	8.99	47.69
T0895-D1	T0895	129	91.67	37.50	0.39	9.08	36.04	0.35	9.99	36.46
T0896-D1	T0896	486	52.94	17.65	0.26	12.33	25.29	0.22	13.08	24.13
T0896-D2	T0896	486	60.00	7.50	0.26	19.95	16.50	0.24	19.26	14.88
T0896-D3	T0896	486	6.25	0.00	0.18	23.78	11.65	0.14	23.46	9.94
T0897-D1	T0897	285	25.00	10.71	0.23	14.20	17.75	0.22	15.01	16.67
T0897-D2	T0897	285	60.00	12.00	0.27	13.81	23.39	0.22	12.79	16.94
T0898-D1	T0898	169	38.10	33.33	0.43	8.42	39.15	0.40	7.34	39.39
T0898-D2	T0898	169	81.82	27.27	0.31	11.18	36.82	0.27	8.99	34.09
T0899-D1	T0899	423	80.77	84.62	0.39	12.89	22.01	0.31	18.14	14.48
T0899-D2	T0899	423	72.22	66.67	0.32	13.97	32.10	0.25	16.01	24.43
T0900-D1	T0900	106	50.00	35.00	0.26	15.89	23.04	0.24	14.59	22.55
T0901-D1	T0901	328	93.33	93.33	0.59	7.69	39.46	0.59	6.44	38.90
T0901-D2	T0901	328	92.86	42.86	0.27	10.80	30.00	0.22	11.07	26.07
T0902-D1	T0902	315	97.83	97.83	0.57	8.86	38.31	0.50	9.84	32.36
T0903-D1	T0903	382	100.00	98.46	0.64	6.01	39.81	0.54	9.48	34.57
T0904-D1	T0904	341	40.00	28.00	0.42	10.34	25.10	0.37	13.63	21.51
T0905-D1	T0905	353	95.83	95.83	0.48	13.44	29.75	0.41	10.82	25.72
T0905-D2	T0905	353	84.62	76.92	0.33	9.74	39.77	0.25	11.00	30.68
T0909-D1	T0909	340	20.00	12.31	0.22	19.36	7.96	0.20	20.08	6.83
T0911-D1	T0911	445	89.02	89.02	0.76	10.20	50.92	0.73	8.38	46.38
T0912-D1	T0912	624	96.39	95.18	0.63	14.14	36.78	0.55	17.17	29.35
T0912-D2	T0912	624	100.00	100.00	0.55	3.96	56.63	0.46	5.09	50.60
T0912-D3	T0912	624	95.24	71.43	0.35	11.86	32.52	0.27	12.20	23.79
T0913-D1	T0913	386	88.24	82.35	0.59	8.33	31.66	0.55	8.94	28.77
T0914-D1	T0914	337	71.88	34.38	0.29	14.52	20.57	0.29	14.52	20.57
T0914-D2	T0914	337	56.25	15.62	0.27	18.34	20.22	0.24	15.39	19.29
T0915-D1	T0915	161	61.29	41.94	0.61	4.62	48.86	0.46	7.99	34.74
T0917-D1	T0917	409	100.00	100.00	0.88	2.87	64.00	0.86	3.67	62.02
T0918-D1	T0918	546	86.36	81.82	0.46	13.20	41.90	0.37	9.21	33.80
T0918-D2	T0918	546	96.00	100.00	0.49	7.15	41.26	0.46	9.00	36.99
T0918-D3	T0918	546	83.33	83.33	0.41	12.86	33.90	0.37	11.99	30.93
T0920-D1	T0920	568	98.44	98.44	0.67	6.28	39.95	0.46	14.74	29.67
T0920-D2	T0920	568	95.45	90.91	0.54	10.08	36.87	0.51	9.63	33.79
T0921-D1	T0921	149	89.29	85.71	0.40	9.64	30.80	0.36	10.40	28.99
T0922-D1	T0922	96	73.33	46.67	0.58	5.90	58.78	0.54	5.83	56.08
T0923-D1	T0923	409	64.91	19.30	0.27	17.00	13.29	0.23	18.30	10.93
T0928-D1	T0928	388	100.00	86.76	0.68	6.43	40.62	0.63	8.20	38.05
T0941-D1	T0941	470	42.65	1.47	0.25	22.27	10.78	0.25	22.74	9.97
T0942-D1	T0942	487	100.00	100.00	0.77	3.63	64.60	0.77	3.63	64.60
T0942-D2	T0942	487	100.00	76.74	0.70	5.18	54.32	0.62	7.51	46.96
T0943-D1	T0943	563	91.67	66.67	0.51	8.08	55.65	0.42	9.11	45.97
T0943-D2	T0943	563	97.75	97.75	0.55	12.21	27.46	0.54	12.60	27.18
T0944-D1	T0944	277	100.00	96.08	0.72	5.70	50.89	0.69	6.13	48.22
T0945-D1	T0945	409	100.00	98.67	0.67	9.42	44.40	0.65	9.55	43.00
T0946-D1	T0946	292	87.50	93.75	0.39	8.00	39.69	0.32	10.04	32.50

Domain	Target	L_{target}	Contact Precision Top L/5		Best of 200 (Models)			Best of 5 (Models)		
			SR + MR + LR	Long-range	TM-score	RMSD	GDTTS	TM-score	RMSD	GDTTS
T0946-D2	T0946	292	92.86	92.86	0.66	8.74	50.83	0.63	8.71	46.58
T0947-D1	T0947	220	97.14	100.00	0.51	11.24	37.86	0.50	15.50	38.57
T0948-D1	T0948	166	53.33	56.67	0.50	9.88	38.93	0.39	11.89	32.21
**Average**			**71.37**	**58.64**	**0.46**	**10.84**	**37.78**	**0.41**	**11.55**	**33.63**
